# QCM-D Investigations on Cholesterol–DNA Tethering of Liposomes to Microbubbles for Therapy

**DOI:** 10.1021/acs.jpcb.2c07256

**Published:** 2023-03-14

**Authors:** Fern J. Armistead, Damien V. B. Batchelor, Benjamin R. G. Johnson, Stephen D. Evans

**Affiliations:** Molecular and Nanoscale Physics Group, School of Physics and Astronomy, University of Leeds, Leeds LS2 9JT, United Kingdom

## Abstract

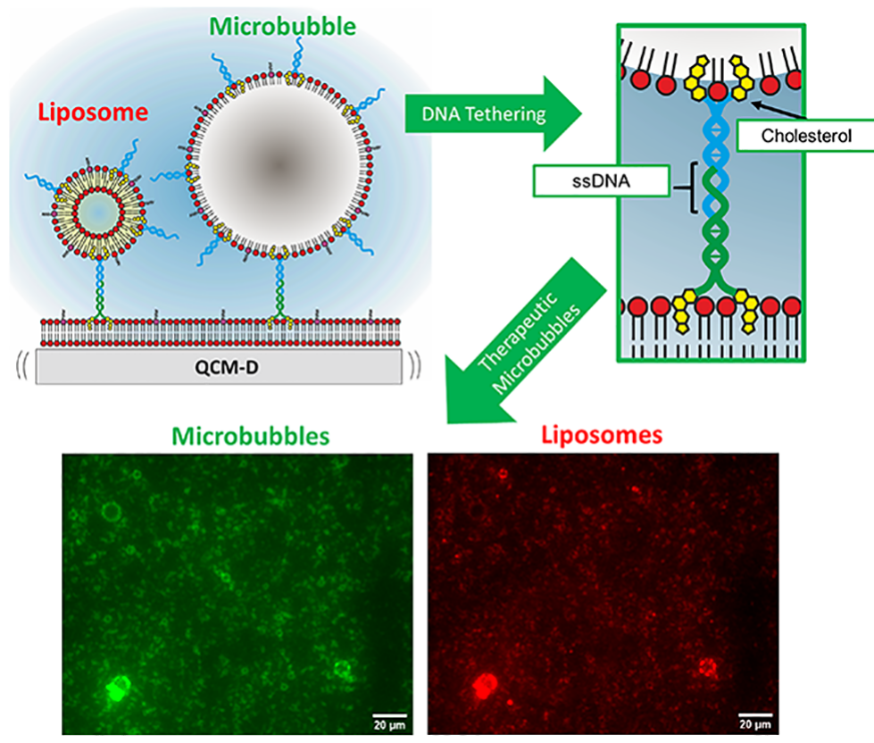

Lipid-shelled microbubbles (MBs) offer potential as theranostic agents, capable of providing both contrast enhancement in ultrasound imaging as well as a route for triggered drug release and improved localized drug delivery. A common motif in the design of such therapeutic vehicles is the attachment of the drug carrier, often in the form of liposomes, to the microbubble. Traditionally, such attachments have been based around biotin–streptavidin and maleimide–PDP chemistries. Comparatively, the use of DNA–lipid tethers offers potential advantage. First, their specificity permits the construction of more complex architectures that might include bespoke combinations of different drug-loaded liposomes and/or targeting groups, such as affimers or antibodies. Second, the use of dual-lipid tether strategies should increase the strength of the individual tethers tethering the liposomes to the bubbles. The ability of cholesterol–DNA (cDNA) tethers for conjugation of liposomes to supported lipid bilayers has previously been demonstrated. For in vivo applications, bubbles and liposomes often contain a proportion of polyethylene glycol (PEG) to promote stealth-like properties and increase lifetimes. However, the associated steric effects may hinder tethering of the drug payload. We show that while the presence of PEG reduced the tethering affinity, cDNA can still be used for the attachment of liposomes to a supported lipid bilayer (SLB) as measured via QCM-D. Importantly, we show, for the first time, that QCM-D can be used to study the tethering of microbubbles to SLBs using cDNA, signified by a decrease in the magnitude of the frequency shift compared to liposomes alone due to the reduced density of the MBs. We then replicate this tethering interaction in the bulk and observe attachment of liposomes to the shell of a central MB and hence formation of a model therapeutic microbubble.

## 1 Introduction

Lipid-based particles are currently used clinically for both diagnostic and therapeutic purposes. For example, microbubbles (MBs) are lipid-shelled, gas-filled bubbles, typically between 1 and 10 μm in diameter and are used routinely for contrast enhanced ultrasound (CEUS).^1^ In combination with ultrasound, MBs can also locally increase intracellular drug uptake either through codelivery of the drug and MB^2,3^ or by attachment of a therapeutic payload to the MB shell. Payloads can be directly attached or incorporated into the MB shell (hydrophobic^4,5^ and electrostatic^6,7^ interactions, chemical linkers,^8^ such as click chemistry,^9^ oil layers^10,11^) or by the attachment of drug-loaded liposomes.^12,13^ Lipid nanoparticles, such as liposomes, which consist of an encasing lipid bilayer and an aqueous core, are already used clinically to deliver chemotherapy drugs such as Doxorubicin and Irinotecan,^14−16^ reducing systemic toxicity. The attachment of liposomes to the MB shell is typically performed using biotin–streptavidin or maleimide–thiol interactions, utilizing lipids with a PEGylated headgroup,^17,18^ and an applied ultrasound trigger can then be used to enhance delivery and uptake of the drug payload.^19,20^

However, these methods of attachment provide little control over the specificity of the chemical interactions between particles, which can lead to agglomeration as well as requiring additional wash steps to remove unincorporated material.^21^ Further, as these reactive groups are commonly conjugated to the lipid head groups, they can also be easily removed from the lipid membrane.

Boxer et al. were the first to demonstrate the ability of cholesterol-based DNA tethers (cDNA) to spontaneously incorporate into lipid membranes, providing a mechanism to attach liposomes to supported lipid bilayers (SLB).^22^ These tethers consist of single-strand DNA (ssDNA) attached to a cholesterol molecule, which enables spontaneous incorporation within lipid bilayers via hydrophobic interactions. The base sequences of the cDNA can be chosen to allow tethering of liposomes to an SLB containing a complementary ssDNA sequence. cDNA can also be used to induce membrane fusion and content mixing, for either liposome*–*liposomes or liposome–SLB, via a zipper-like interaction.^23−25^ While a single cholesterol anchor is relatively weak (*k*_off_ ≈ 6 × 10^–4^ s^–1^),^26^ Höök et al. demonstrated nearly irreversible coupling using a bivalent cholesterol anchor,^27^ achieved by coupling of 15-mer and 30-mer ssDNA, each with a single cholesterol anchor in the 3′- and 5′-ends, respectively.

Here, we propose the use of cDNA tethers to produce therapeutic MBs (i.e., MBs with therapeutic liposomes attached to the shell). This cDNA tethering strategy is advantageous compared to traditional conjugation methods as (i) chemically modified lipids do not need to be introduced to the system, (ii) it is more specific (arbitrary ssDNA sequences can be designed and combined), and (iii) the method is faster with increased ease of use (i.e., cDNA can be added after liposome or MB production).

Incorporation of cDNA into a SLB and the subsequent attachment of liposomes can be quantified using quartz crystal microbalance with dissipation (QCM-D), in which a SiO_2_ crystal is driven at resonance. QCM-D allows measurement of the coupled mass to the crystal, in which the formation of an SLB on the surface is denoted by a characteristic decrease in frequency. The sensitivity of QCM-D (∼1 ng/cm^2^)^28,29^ allows for monitoring of the adsorption of proteins onto a biotinylated SLB,^30^ upon which biotinylated liposomes can also be attached.^31,32^ Höök et al.^27^ also utilized QCM-D to monitor the spontaneous incorporation of cDNA (both mono- and bivalent) into the SLB.

Therapeutic lipid particles (e.g., liposomes, lipid nanoparticles, microbubbles) commonly contain a small proportion of polyethylene glycol (PEG) to enhance the lifetime in vivo by providing “stealth-like” properties and reducing a subsequent immune response. In this paper, first, we investigate the influence of the presence of PEG on cDNA-mediated tethering between liposomes and an SLB, both containing PEG. We then demonstrate the ability of cDNA to spontaneously incorporate within the lipid monolayer of a MB and use this to tether MBs to an SLB. Importantly, we show that we can discriminate between the attachment of MBs, compared to liposomes (of which both are present in a MB sample), to the SLB due to the reduced density of the MB gas core. We then demonstrate the ability to replicate this interaction in a bulk solution and use cDNA to tether model therapeutic liposomes to a central MB and formation of a dual therapeutic and diagnostic MB. This opens the possibility for selective attachment of different therapeutics to a single MB, with additional specificity provided by cDNA and allowing for fine control over the number of liposomes attached to each MB.

## 2 Methods

### 2.1 QCM-D

Quartz crystal microbalance with dissipation (QCM-D) was performed using the Q-Sense E4 instrument (Biolin Scientific) with commercially available quartz crystal sensors with a center frequency of 4.97 MHz (Biolin Scientific). QCM-D sensors were cleaned before each experiment by sonication for 30 min in 1% SDS (sodium dodecyl sulfate), followed by Milli-Q (MQ) water, and then isopropanol. Crystals were then dried under a nitrogen stream and further cleaned in a UV/ozone oven for 30 min.

Once cleaned, the sensors were secured inside the flow chambers in the QCM-D instrument. At the start of each experiment, degassed MQ was flowed at 100 μL/min until a steady baseline was achieved for frequency and dissipation, where several harmonics were found (typically 3rd to 11th). Measurements were then restarted to successfully calibrate frequency and dissipation to 0 at the start of each experiment. Samples were then sequentially flowed into the chambers at 100 μL/min, where changes to frequency and dissipation were indicative of adhesion of mass to the QCM-D sensors.

### 2.2 Liposome Production

Liposomes of the following composition were prepared at a total lipid concentration of 0.5 mg/mL: (1) POPC (1-palmitoyl-2-oleoyl-*sn*-glycero-3-phosphocholine), (2) 99:1 molar ratio of POPC:DSPE-PEG2000 (1,2-distearoyl-*sn*-glycero-3-phosphoethanolamine-*N*-[methoxy(polyethylene glycol)-2000]), (3) 95:5 molar ratio of DPPC:DSPE-PEG2000, and (4) 63:32:5:0.5 molar ratio of DSPC:cholesterol:DSPE-PEG2000:DHPE-TexasRed. Lipid stocks were dissolved in 1:1 chloroform and methanol and were dried under nitrogen to remove the solvent before resuspension in phosphate-buffered saline (PBS). The dried lipid film was resuspended by ∼1 min vortexing. Solutions were then tip sonicated for 30 min, resulting in a clear liposome-containing solution. For QCM-D experiments involving bilayer formation and liposome tethering, liposome solutions were diluted in PBS to 0.2 mg/mL.

### 2.3 Microbubble Production and Characterization

Microbubbles (MBs) were prepared from a 95:5 molar ratio of DPPC (1,2-dipalmitoyl-*sn*-glycero-3-phosphocholine) and DPSE-PEG2000 at a total lipid concentration of 2 mg/mL (Avanti Polar Lipids, AL, USA). Lipid stocks were dissolved in 1:1 chloroform and methanol and were dried under nitrogen to remove the solvent before resuspension in phosphate-buffered saline (PBS). The dried lipid film was resuspended by heating at 45 °C for ∼15 min followed by ∼1 min of vortexing, until a cloudy solution was achieved. To produce the initial bubble solution, 1 mL of liposomes solution (2 mg/mL) was added to a 1.5 mL glass vial, and the solution and vial headspace were saturated with C_3_F_8_ (perfluoropropane) gas, maintaining a gas pressure of 300 mbar for 2 min. Gas flow was controlled using a p-pump (Mitos P-pumps, Dolomite, UK) and a PC using the Dolomite Flow Control Centre.^33^ The vial lid was then replaced and sealed with parafilm prior to mechanical agitation for 45 s (VialMix, Bristol Myers Squibb, US).

#### 2.3.1 Bright-Field and Fluorescence Microscopies

Bright-field microscopy was used to characterize the size and concentration of the MB population. A 30 μL volume of sample was introduced into a 50 μm depth chamber on a glass slide, and MBs were allowed to rise for 5 min to ensure they were all in the same focal plane.^34^ An inverted microscope (Nikon 90i, Japan) was used to image the bubbles with a 40× objective (NA = 0.6), and a CCD camera (DS-Fil 5Mega pixel, Nikon, Japan) was used to take 10 images for each sample. Microbubble size and concentration were analyzed using a custom MATLAB script.^35^ Fluorescence microscopy was also used to observe fluorescence from MBs and liposomes using the same optical setup but with the addition of FITC (Ex 467–498 nm, Em 513–556 nm) and TexasRed (Ex 542–582 nm, Em 604–644 nm) filter cubes.

### 2.4 Cholesterol–DNA

**Figure 1:**
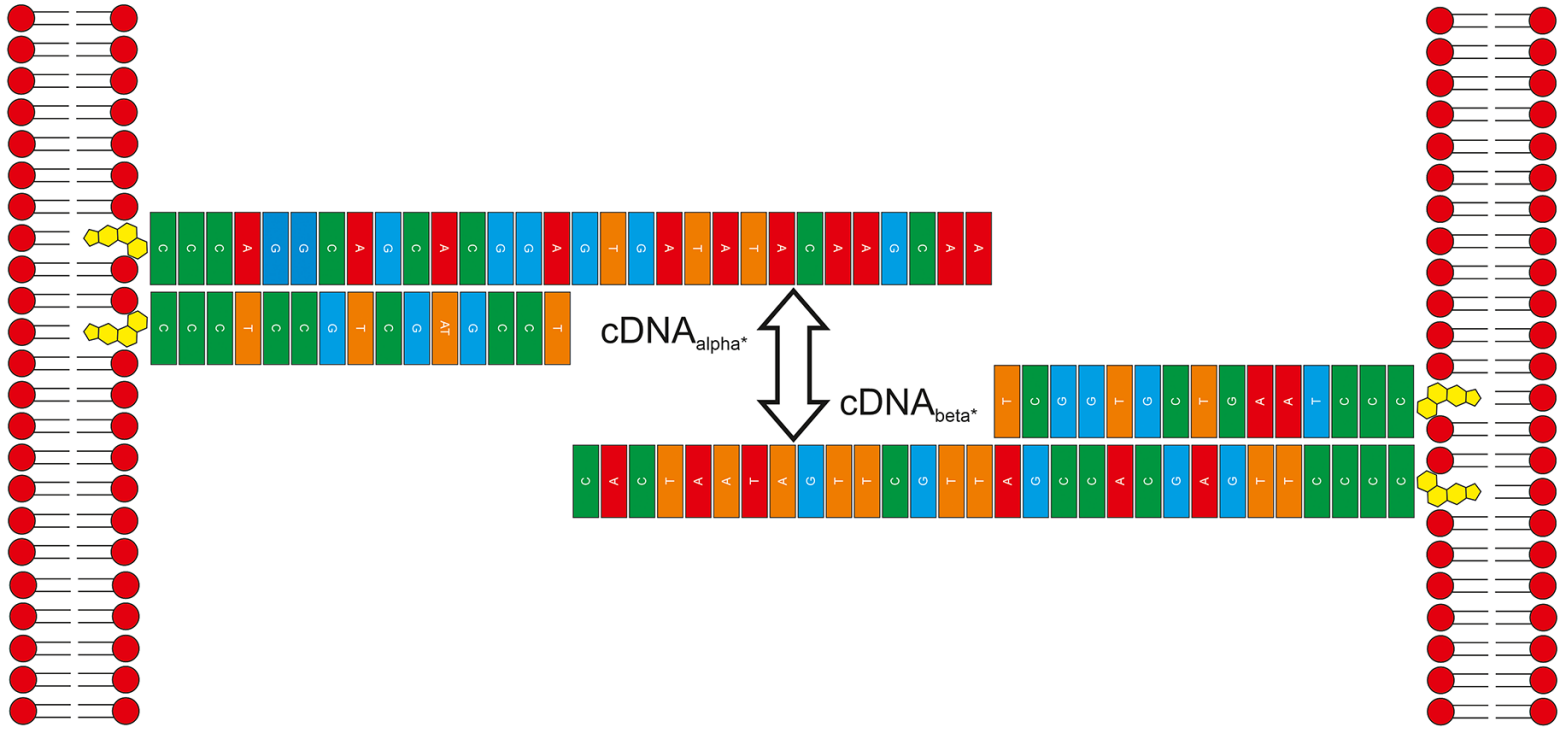
Schematic describing the cholesterol-functionalized ssDNA tethering mechanism used in the study. A 15-mer (cDNA_alpha′_/cDNA_beta′_) was bound to a 30-mer (cDNA_alpha_/cDNA_beta_) using complementary base sequences creating a double-cholesterol anchoring point at one end. The remaining 15 unbound bases of each double-anchored conjugate (cDNA_alpha*_/cDNA_beta*_) then had complementary sequences resulting in tethering.

All cholesterol–DNA (cDNA) derivatives were purchased from Eurogentec, Belgium, and stocks were prepared in TE buffer (10 mM TRIS, 1 mM ethylenediaminetetraacetate, pH 8) at a stock concentration of 40 μM. Table 1 shows the cDNA sequences used in the study. The role of the 15-mer cDNA_alpha′_ was to bind to the 30-mer cDNA_alpha_, creating a double-cholesterol anchoring point and leaving the remaining 15 bases of the cDNA_alpha_ available for tethering to a complementary strand. The double-cholesterol conjugate of cDNA_alpha_ and cDNA_alpha′_ will be referred to as cDNA_alpha*_ and was formed by mixing equal amounts of cDNA_alpha_ and cDNA_alpha′_ for >10 min. Complementary to this, cDNA_beta_ and cDNA_beta′_ are conjugated in the same way and referred to as cDNA_beta*_. The mixing of cDNA_alpha*_ and cDNA_beta*_ thus results in tethering of the available and complementary chain of 15 base pairs, leaving double-cholesterol anchoring points on both ends which spontaneously insert into lipid monolayers/bilayers. The schematic in Figure 1 demonstrates how the cDNA sequences bind together.

**Table 1:** List of Cholesterol-Functionalized DNA Sequences Used in the Study

name	sequence
cDNA_alpha_	5′-AAC-GAA-CAT-ATA-GTG-AGGCAC-GAC-GGA-CCC-cholesterol-3′
cDNA_alpha′_	5′-cholesterol-CCC-TCC-GTC-GTG-CCT-3′
cDNA_beta_	5′-CAC-TAT-ATG-TTC-GTT-AGC-CAC-GAG-TTC-CCC-cholesterol-3′
cDNA_beta′_	5′-cholesterol-CCC-GAA-CTC-GTG-GCT-3′

### 2.5 Ultrasound

A single-element high-intensity focused ultrasound (HIFU) transducer was used for destruction of microbubbles. The HIFU transducer (H-102, Sonic Concepts, USA) had a center frequency of 1.1 MHz and was connected to a +53 dB power amplifier through an impedance matching circuit. Sinusoidal burst cycles were provided to the transducer using a computer-controlled function generator (33220A, Agilent, USA), and a membrane hydrophone (Precision Acoustic Ltd., Dorchester, UK) was used to measure the free-field pressure with a 400 μm sensitive element, calibrated by the National Physics Laboratory (Middlesex, UK). Stated pressures are based on free-field calibrations, where error was ±0.1 MPa.

MB samples were contained in an 1.5 mL centrifuge tube and coupled to the HIFU transducer via a coupling cone containing degassed MQ water, such that MBs were positioned in the focal point of the ultrasound field (focal length = 13.5 mm, focal width (fwhm) = 1.33 mm, *f* number = 0.98).^36^ Samples were exposed to HIFU for 5 s pulses repeated 5 times using a 300 mV peak to peak voltage (equivalent to a peak negative pressure of 5.8 MPa) with 1% duty cycle and 1 kHz pulse repetition frequency. Initially cloudy samples were more visibly transparent after HIFU exposure. Optical imaging was then used to confirm a significant reduction in MB concentration.

### 2.6 cDNA Tethering Protocols

cDNA_alpha*_ and cDNA_beta*_ conjugates were formed by incubation of equal amounts of cDNA_alpha_ and cDNA_alpha′_ or cDNA_beta_ and cDNA_beta′_, respectively, for >10 min.

#### 2.6.1 Liposome–Bilayer

cDNA was added to liposome solutions before bilayer formation. POPC or DPPC liposomes were diluted in PBS to 0.2 mg/mL; cDNA_alpha*_ was then added at a final concentration of 80 nM. Bilayers were then formed on the quartz QCM-D sensors. Complementary liposomes were prepared simultaneously. Liposome solutions were diluted to 0.2 mg/mL in PBS, and cDNA_beta*_ was added at a final concentration of 80 nM. Complementary liposomes were then tethered to the SLB, which was characterized using QCM-D.

#### 2.6.2 Bubble–Bilayer

cDNA_alpha*_ was incorporated into POPC bilayers as previously described at a final concentration of 80 nM. cDNA_beta*_ was incorporated into microbubble solution after production using mechanical agitation and then diluted 10× in PBS from their stock concentration.

#### 2.6.3 Bubble–Liposome

cDNA_beta*_ was incorporated into model therapeutic liposomes, which consisted of DSPC, cholesterol, DSPE-PEG2000, and DHPE-TexasRed, as previously described,^20,37−40^ with liposomes at a final concentration of 0.5 mg/mL. To integrate cDNA_beta*_ into liposomes, 10 μL of cDNA_beta*_ at a concentration of 1.3 μM was mixed with 150 μL of liposomes and left for 10 min. Two separate methods of incorporating cDNA_alpha*_ into MBs were investigated, by adding to the lipid solution either prior to MB production or after mechanical agitation and MB formation. For the scenario where cDNA was incorporated pre-MB production, 10 μL of cDNA_alpha*_ (2 μM) was added to 1 mL of MB lipid solution (2 mg/mL) and incubated for 10 min. MBs were then produced as described in section 2.3. MBs were then diluted by a factor of 10 in PBS, and 100 μL of cDNA_alpha*_-MBs added to 50 μL of cDNA_beta*_ liposomes. For the scenario where cDNA was incorporated after MB production, 10 μL of cDNA_alpha*_ was added to 200 μL of MBs that had already been diluted by a factor of 10 in PBS and left for 10 min. These MBs were then combined with 100 μL of cDNA_beta*_ to promote tethering.

## 3 Results and Discussion

### 3.1 Liposome/Bilayer Tethering

**Figure 2:**
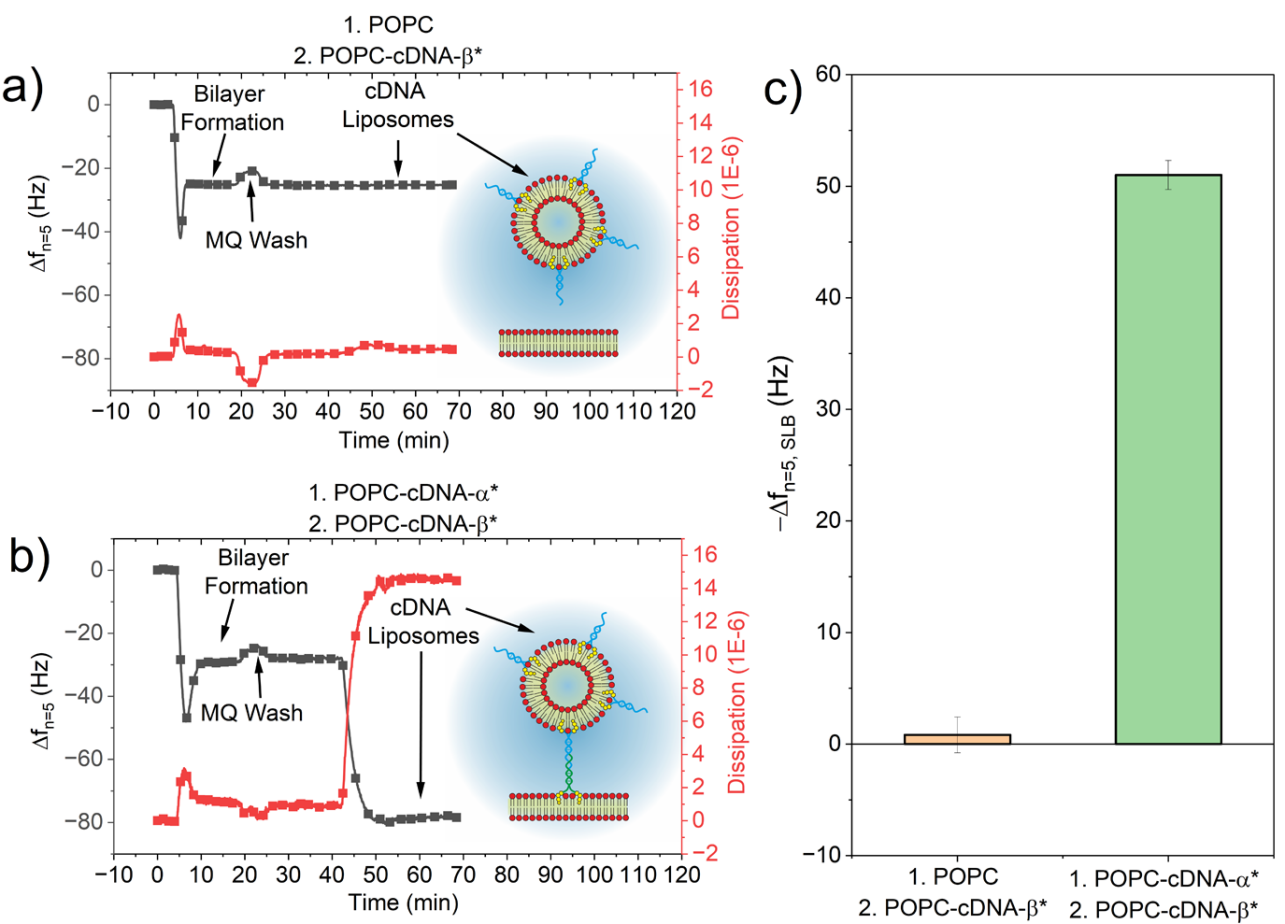
QCM-D frequency (black) and dissipation (red) data investigating the tethering affinity between POPC-cDNA-_beta*_ liposomes and an SLB consisting of either (a) POPC only or (b) POPC-cDNA_alpha*_, in which alpha* and beta* denote complementary DNA strands. Note that the schematics are not to scale; vesicles are typically ∼80 nm in diameter, while a typical 30-mer is about 20 nm in length, and the bilayer is approximately 4 nm thick. (c) The frequency shift from the SLB after incubation with POPC-cDNA_beta*_ liposomes to a POPC only or POPC-cDNA-_alpha*_ SLB.

QCM-D was used to quantify the interactions between cholesterol–DNA (cDNA) and lipid membranes, as the change in resonance frequency of the QCM-D crystal is directly proportional to the coupled mass. Dissipation of the system is also monitored, defined as the sum of all energy losses per oscillation cycle of the crystal, after the drive generator output is stopped, leading to exponential decay of the sensor oscillation. Changes in dissipation provide information on the viscoelastic properties of the system, as a “soft” more viscoelastic film has a slower decay and hence increased dissipation, compared to a more rigid film,^40^ which can also be attributed to the deposition or attachment of flexible layer particles (i.e., liposomes).^41^ cDNA tethers were prepared with a double-cholesterol anchor, shown by Höök et al. to increase the strength of coupling to the lipid membranes.^27^ This was achieved by hybridization between a 15-mer ss-DNA and a 30-mer ss-DNA, both modified with cholesterol in the 3′- and 5′-ends, respectively (Figure 2). The cDNA was incorporated into liposomes by incubation for 10 min. SLBs (either with or without cDNA) were formed by incubation of liposomes on the QCM-D crystal.

First, we demonstrated the ability of cDNA to mediate liposome attachment to SLBs. Figure 2a shows QCM-D data for the formation of a POPC only bilayer, in which both the frequency shift from the stable baseline (Δ*f*) and the dissipation of the system is shown. Evidence of SLB formation on the quartz crystal is signified by the characteristic decrease in resonance frequency, *f*, during which POPC liposomes are adsorbed onto the surface, followed by an increase in *f* as liposomes rupture and form a SLB.^42^ The final Δ*f* after POPC SLB formation and a PBS-MQ-PBS wash was a characteristic value of ca. −26 Hz.^31^ This control SLB, containing no cDNA, was then incubated with liposomes containing POPC-cDNA_beta*._ No frequency change and a negligible change in dissipation (<0.5 × 10^–6^) was observed, indicative of no cDNA-based tethering of the liposomes to the SLB.

Figure 2b shows the formation of an SLB containing POPC–cDNA_alpha*_ in which Δ*f* ≈ −27 Hz. This slight increase in magnitude may be attributed to the additional mass of the SLB upon incorporation of cDNA_alpha*_. Further incubation with POPC–cDNA_beta*_ liposomes led to an additional decrease from the SLB formation *f* of ∼50 Hz, providing evidence of successful cDNA-based tethering of liposomes to the SLB. A large increase in dissipation of the system of ∼14 × 10^–6^ was also observed upon liposome tethering due to additional damping and energy loss associated with liposome tethering.^43^ The summary of changes in frequency from the SLB induced by liposome tethering (Δ*f*_*n*=5,SLB_) is shown in Figure 2c.

### 3.2 Influence of PEG on cDNA Tethering Affinity

**Figure 3:**
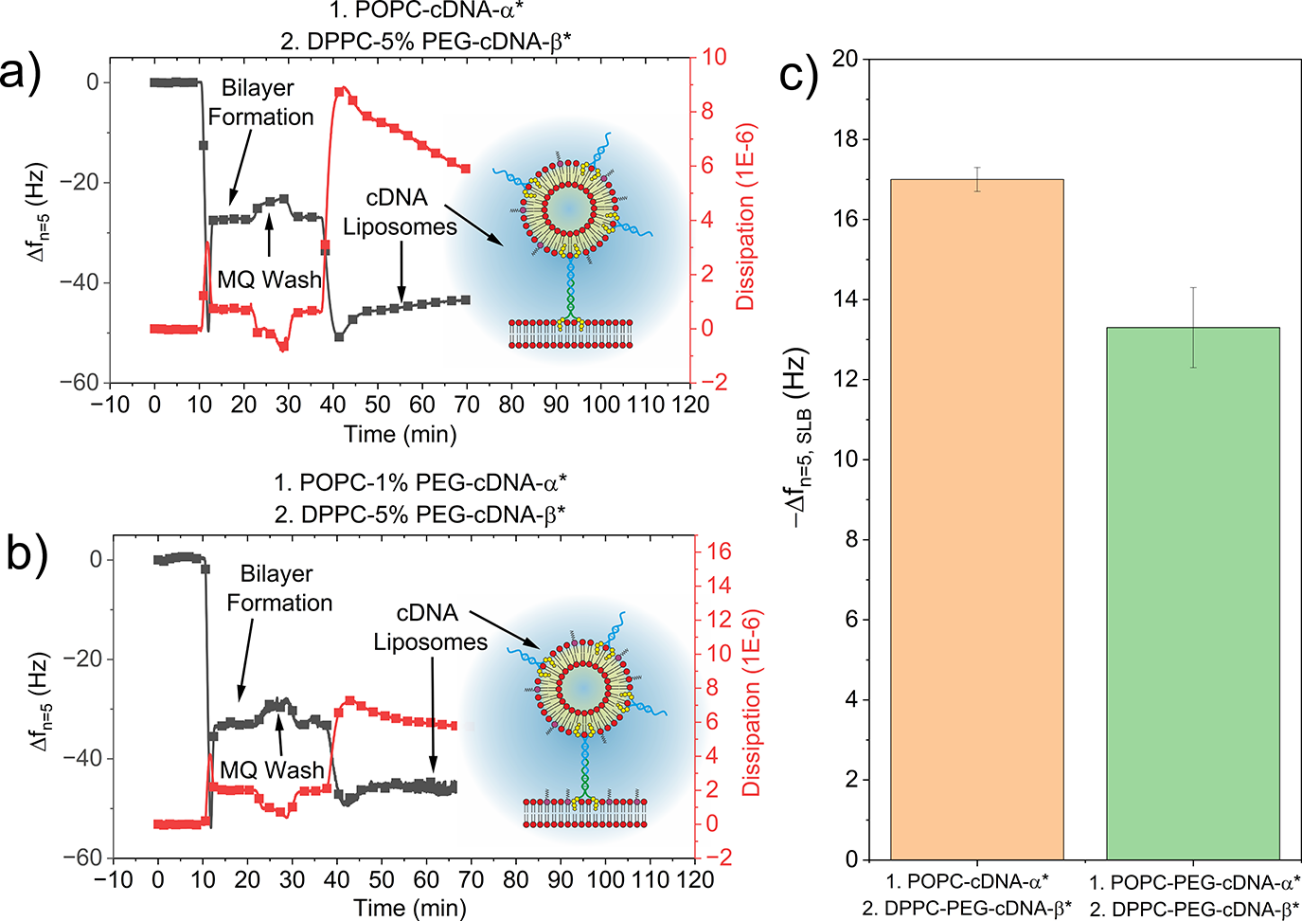
QCM-D frequency (black) and dissipation (red) data investigating the tethering affinity between DPPC-5% PEG-cDNA-_beta*_ liposomes and an SLB consisting of either (a) POPC-cDNA_alpha*_ only or (b) POPC-1% PEG-cDNA_alpha*_, in which alpha* and beta* denote complementary DNA strands. (c) Summary of the frequency shift from the SLB for each condition.

Therapeutic microbubbles, nanobubbles, and liposomes commonly contain a proportion of polyethylene glycol (PEG) in their shell.^20,37,38,44,45^ This PEG layer can be used to enhance the in vivo lifetime and provide additional functionalization.^17,46^ However, the steric effects associated with this may hinder the ability of cDNA to function effectively. Here, we investigate how the presence of PEG influences cDNA-based tethering of liposomes to SLBs. As a model system for the tethering between liposomes and therapeutic bubbles, we observed tethering between liposomes and a POPC SLB with or without PEG. Liposomes consisted of 95% DPPC and 5% DSPE-PEG2000 prior to incorporation of cDNA_beta*_ and the SLB either POPC only or with the incorporation of 1% DSPE-PEG2000 shown in the inset in Figure 3a and 3b, respectively. It should be noted here that the change of the main lipid component of the liposomes from POPC to DPPC (i.e., from an unsaturated to a saturated hydrocarbon tail) was made to provide a closer mimic to that of the therapeutic vehicle.

Data in Figure 3a shows the formation of a POPC-SLB, indicated by a Δ*f* of ca. −26 Hz. Incubation with DPPC-5% PEG-cDNA_beta*_ liposomes, followed by washing, led to a further frequency shift of ca. −17 Hz, suggesting successful liposome attachment but to a lesser extent than that in the absence of PEG (Δ*f* ≈ −50 Hz, Figure 2b). This experiment was repeated in which PEG was present in both the cDNA_beta*_ liposomes and the cDNA_alpha*_ SLB (Figure 3b). The proportion of PEG (1%) within the SLB was chosen as the highest molar percentage that enabled successful liposomal rupture and stable SLB formation on the SiO_2_ crystal. Upon formation of the SLB, the magnitude of the frequency shift, Δ*f*, was ca. −35 Hz, which can be attributed to the increased mass of the SLB with the inclusion of PEG. Incubation with DPPC-5% PEG-cDNA_beta*_ liposomes followed by washing led to a further frequency shift, Δ*f*_SLB_, of ca. −13 Hz, indicative of successful attachment to the SLB. Again, the magnitude of the frequency shift decreased further, likely due to the presence of PEG in both constructs inhibiting attachment (Figure 3c).

### 3.3 MB–Bilayer Interactions

**Figure 4:**
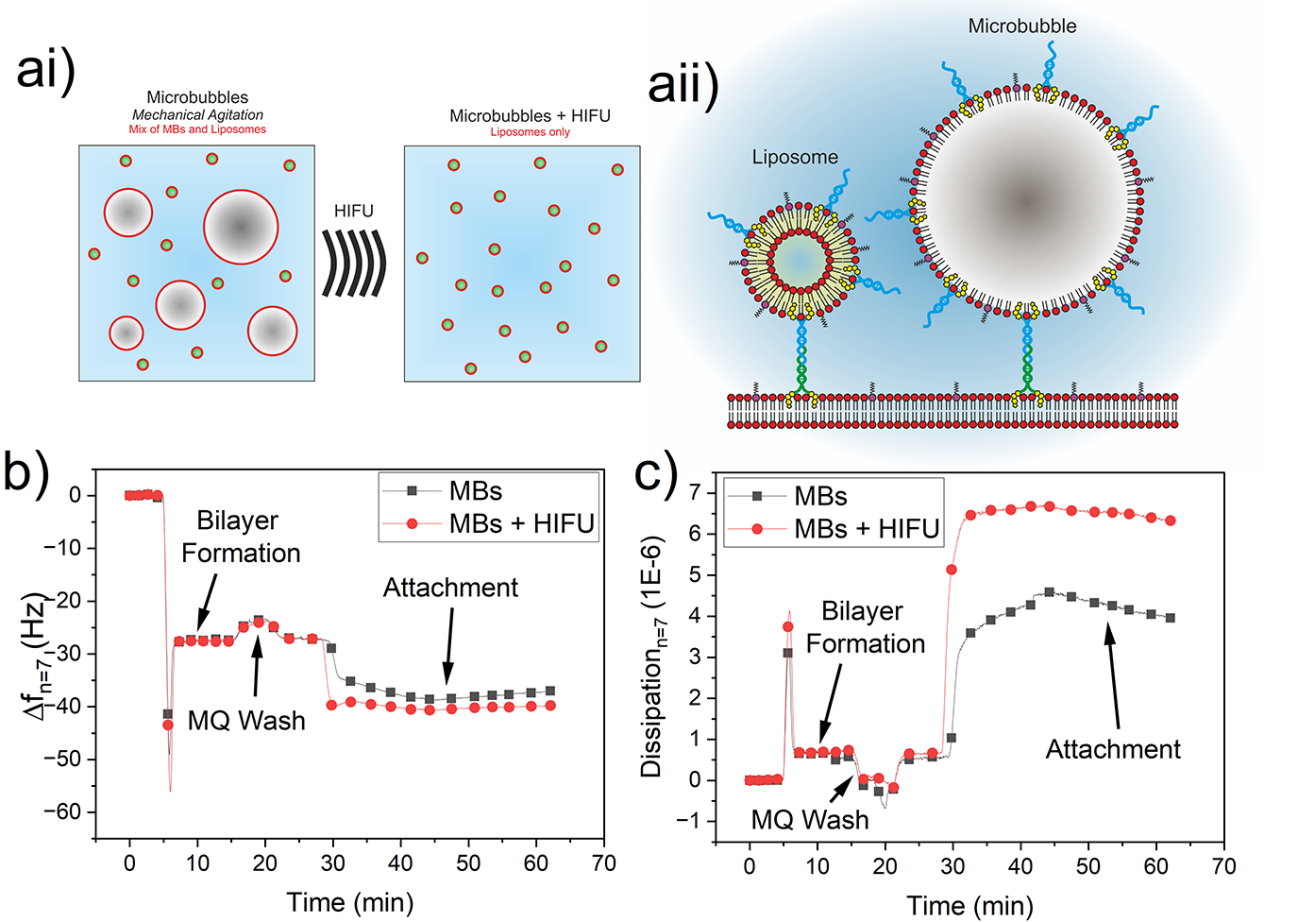
(ai) Liposomes containing DPPC and DSPE-PEG2000 are produced via tip sonication, which are subsequently mechanically agitated to produce a MB sample which contains a mixed population of both gas-cored MBs and aqueous-cored liposomes. The application of HIFU (high-intensity focused ultrasound) can be used to remove gas-cored MBs from the solution. (aii) Schematic showing a comparison between cDNA tethering of a liposome compared to a MB to a SLB. (b and c) QCM-D data assessing the interactions between a microbubble (MB) sample containing cDNA_beta*_ and a POPC-cDNA_alpha*_ SLB showing (b) frequency and (c) dissipation data. MBs denotes a MB sample, and MBs + HIFU denotes the same MB sample that was exposed to high-intensity focused ultrasound (HIFU) to destroy MBs in the sample. All data were collected from the 7th harmonic.

Previous data demonstrates the ability of the cDNA to incorporate within lipid bilayers (either within the liposomes or a SLB formed on SiO_2_) and initiate tethering between liposomes and SLBs. However, lipid-shelled microbubbles (MBs) have a lipid monolayer, as opposed to a lipid bilayer. While it is assumed that the same anchoring mechanisms will apply in the case of MBs, we tested this by attaching MBs to a SLB using cDNA tethers. To investigate this, QCM-D was used to assess the tethering of MBs (95:5 DPPC:DSPE-PEG2000) containing cDNA_beta*,_ to a cDNA_alpha*_-POPC SLB. MB samples contain a mixture of both gas-cored bubbles with a lipid monolayer and excess liposomes with an aqueous core and lipid bilayer. To help distinguish between these two subpopulations, MB samples were treated with high-intensity focused ultrasound (HIFU) to destroy the acoustically active, gas-filled MBs with only aqueous-cored liposomes remaining (Figure 4ai). After HIFU exposure, MB concentration decreased from an initial value of 5 × 10^10^ to 1 × 10^8^ MB/mL (Supplementary Figure 1). MB samples were then diluted 10× from their initial concentration such that the resultant total lipid concentration matched that used in previous sections (0.2 mg/mL). A schematic showing a comparison between cDNA tethering of a liposome and a MB is shown in Figure 4aii.

QCM-D data is shown in Figure 4b and 4c for the two samples (MBs and MBs + HIFU) showing both frequency and dissipation, respectively. In both cases, formation of a cDNA_alpha_-POPC SLB led to a frequency shift, Δ*f*, of ca. −28 Hz. Incubation with the MB sample before HIFU treatment led to a further frequency shift of ∼10 Hz and hence successful tethering of MBs to the SLB. This shift is also noticeably lower in magnitude than that observed for liposomes only (Δ*f* ≈ −17 Hz, Figure 3c), which may be attributed to a reduction in the mass coupled to the QCM-D sensor due to the lower density gas core of MBs compared to liposomes. Tethering of the MB sample after HIFU treatment led to a shift of ∼12 Hz, in which the increase in magnitude again can be attributed to removal of gas-filled MBs from the sample. It should be noted that MB samples likely also contain a proportion of submicrometer bubbles (i.e., nanobubbles)^17,34,36^ that would also be able to interact with the SLB using the cDNA tethers. However, our recent work has shown that the HIFU treatment is also capable of inducing NB destruction.^34^

Interestingly, dissipation data showed the reverse trend in which MBs only had a reduced dissipation (∼4.5 × 10^–6^) than that of MBs + HIFU (∼6.5 × 10^–6^). There are multiple factors that can influence the dissipation of our system, which may lead to changes in dissipation between MBs or liposomes tethered to the SLB. The degree of coupling of the tethered particles to the aqueous phase, primarily through hydrogen-bonding and ionic interactions, can be assumed to be the same for both bubbles and liposomes as particle shell composition would be the same. As MBs are larger than liposomes (∼2 μm compared to ∼100 nm), their increased physical coupling to the aqueous phase would contribute to increased dissipation. However, their increased size will result in an increased number of possible tethering sites per particle, leading to increasing stiffness of attachment to the SLB, reducing dissipation. MBs may dominate the tethering due to their larger size and surface area, and hence, a lesser total number of particles are attached, decreasing dissipation. The stiffness of the tethered particles will also influence dissipation, with MBs having an elastic modulus ∼100× greater than that of liposomes (10^9^ compared to 10^7^ Pa^46−48^), decreasing dissipation. As such, further work is needed to understand the contributions from each of these factors to the dissipation and energy losses of the system.

### 4.4 Therapeutic cDNA Microbubbles

**Figure 5:**
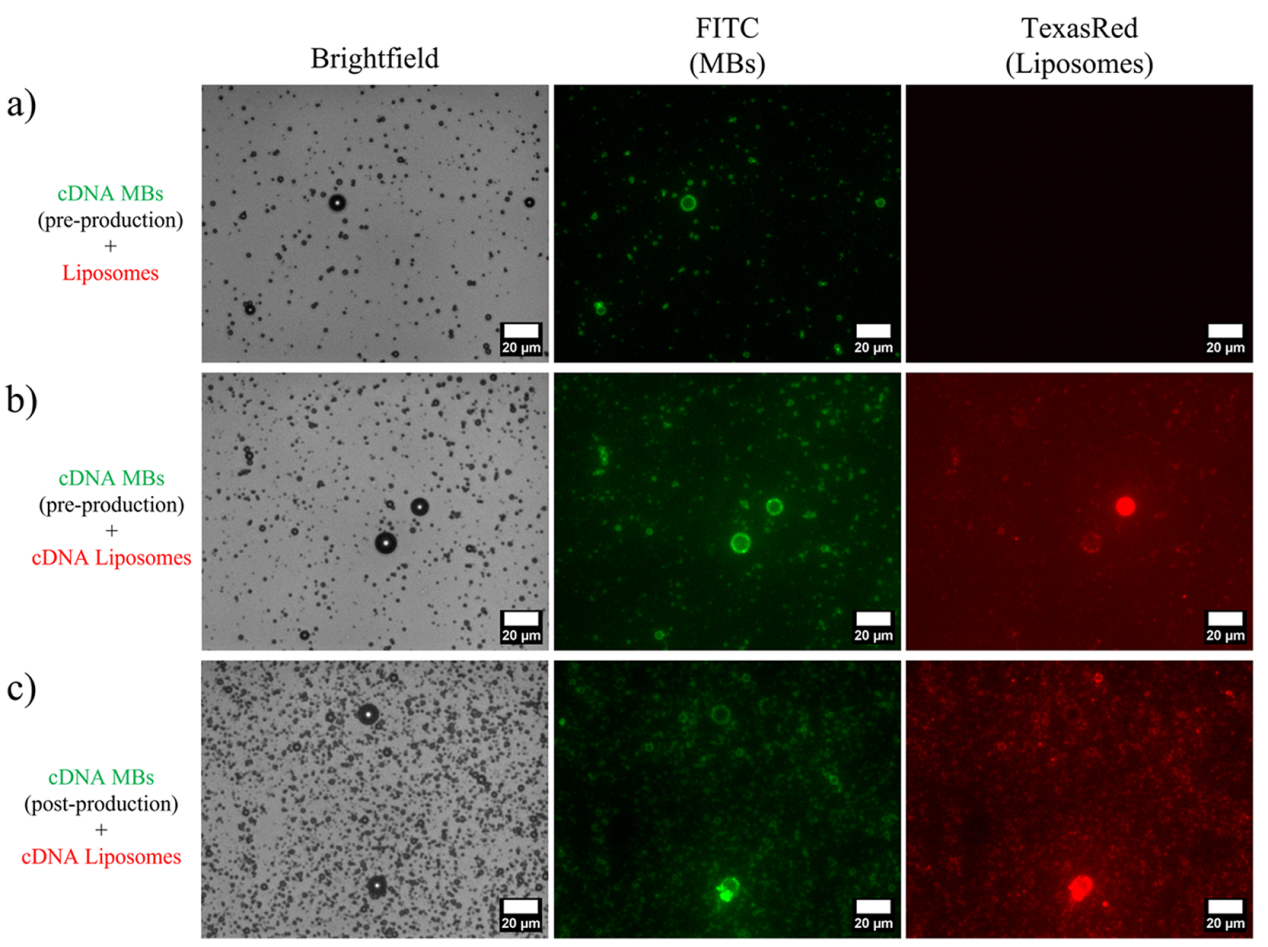
Bright field and fluorescence images of combined MBs (FITC) and liposomes (TexasRed). (a) Control sample of FITC cDNA_beta*_ MBs and TexasRed liposomes (no cDNA), showing no colocalization and hence no liposome conjugation. (b) FITC cDNA_beta*_ MBs (in which cDNA was added pre-MB production) and TexasRed cDNA_alpha*_ liposomes, showing colocalization between fluorescence channels and hence tethering of liposomes to the MB shell. (c) FITC cDNA_beta*_ MBs (in which cDNA was added post-MB production) and TexasRed cDNA_alpha*_ liposomes, in which tethering was observed.

QCM-D data in Figure 4 demonstrated the ability to tether lipid-shelled MBs to a SLB using cDNA. Here, we replicate this interaction in the bulk medium and demonstrate the ability to attach model-therapeutic liposomes to a central MB with cDNA. cDNA_beta*_ was incorporated into the MB shell by addition of cDNA to the liposome precursor solution (95:5 DPPC:DSPE-PEG2000) prior to mechanical agitation. These MBs, which also contained a FITC fluorescent tag, were then incubated in solution with TexasRed-tagged liposomes containing the complementary cDNA_alpha*_ to promote conjugation. Bright-field and fluorescence microscopies were used to locate and identify MBs (FITC) and liposomes (TexasRed) (Figure 5). A control sample in which no cDNA was present in the liposome sample showed no signs of fluorescence colocalization of FITC and TexasRed and hence no cDNA tethering (Figure 5a). Incorporation of cDNA_beta*_ into the MBs prior to their production via mechanical agitation and incubation with cDNA liposomes led to successful tethering (Figure 5b). This process can also be repeated by adding the cDNA_beta*_ to the MB solution after their initial production, which can reduce the quantity, and hence cost, of cDNA required and also allow for precise tailoring of the ratio of cDNA to individual MBs.

## 4 Conclusion

This study demonstrated the potential to use cDNA tethers in the formation of theranostic agents, namely, therapeutic microbubbles (MBs). It was shown that through the use of complementary cDNA strands, model therapeutic liposomes can be tethered to supported lipid bilayers (SLBs) in the presence of polyethylene glycol in both the liposome and the SLB. The ability of cDNA to spontaneously incorporate within the lipid monolayer of gas-cored microbubbles was demonstrated using QCM-D and then used to attach MBs to an SLB. Attachment of MBs to the SLB was distinguished from that of aqueous-cored liposomes by a decrease in the coupled mass to the SLB, due to the decreased density of the MB gas core showing promise for QCM-D as a technique to distinguish bubbles from non-bubbles. QCM-D may also be able to quantify bubble–surface interactions in a model system and help to further understand fundamental interactions between MBs and SLBs.

This interaction was then replicated in the bulk in which model therapeutic liposomes were tethered to MBs, demonstrated via fluorescence colocalization. However, it is yet to be seen how this conjugation method will trigger an immune response and if the quantities of cDNA required would be translatable into a clinical environment. Further work will consist of fundamental investigations into how MBs influence the QCM-D measurement technique.

## Supporting Information Available

Schematic of the HIFU set up used for MB treatment; MB size and concentration before and after HIFU treatment (PDF)
