# Supplementary material for: QCM-D Investigations on Cholesterol–DNA Tethering of Liposomes to Microbubbles for Therapy
Source: J Phys Chem B. 2023 Mar 14;127(11):2466–74. doi: 10.1021/acs.jpcb.2c07256 (PMC10041634; doi:10.1021/acs.jpcb.2c07256)
Supplement: Supplementary file 1 — jp2c07256_si_001.pdf [file jp2c07256_si_001.pdf]

Supplementary Material: QCM-D Investigations on Cholesterol-DNA Tethering of Liposomes to Microbubbles for Therapy

Fern J. Armistead [a,†], Damien V. B. Batchelor [a,†], Benjamin R. G. Johnson [a], Stephen D. Evans [a]*

[a] Molecular and Nanoscale Physics Group, School of Physics and Astronomy, University of Leeds, LS2 9JT, United Kingdom

† indicates authors contributed equally

* Corresponding author: s.d.evans@leeds.ac.uk

KEYWORDS Microbubbles, QCM-D, DNA tethers, Liposomes, Lipid nanoparticles

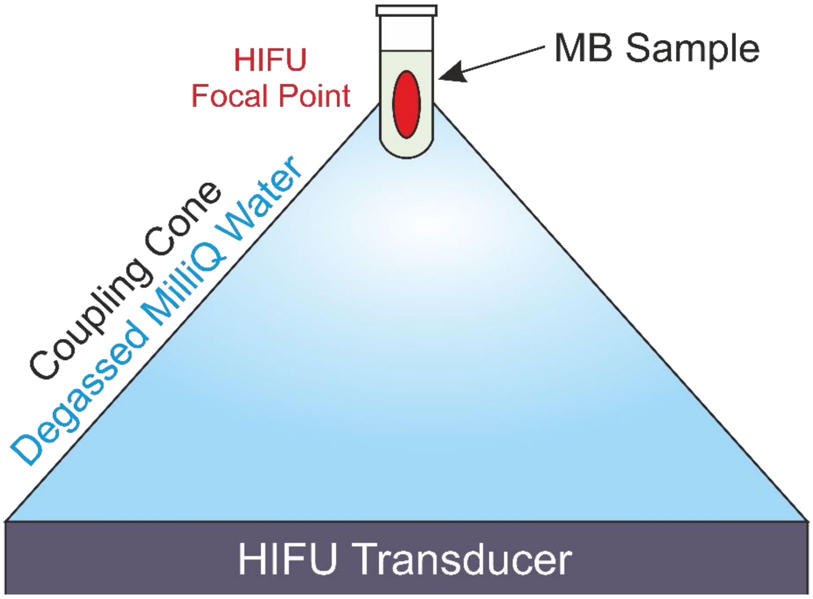

Scheme S1 – Schematic of the high intensity focused ultrasound (HIFU) experimental set up for insonation and destruction of microbubbles (MBs). A HIFU transducer (central frequency = 1.1 MHz, focal length = 13.5 mm, focal width (FWHM) = 1.33 mm, f-number = 0.98) was used to treat a MB sample contained within a 1.5 mL centrifuge tube, situated within the focal point of the ultrasound field coupled using a coupling cone containing degassed MilliQ water. Samples were exposed for 5 x 5 s pulses (peak negative pressure = 5.8 MPa, 1 % duty cycle, 1 kHz pulse repetition frequency) to ensure MB destruction.

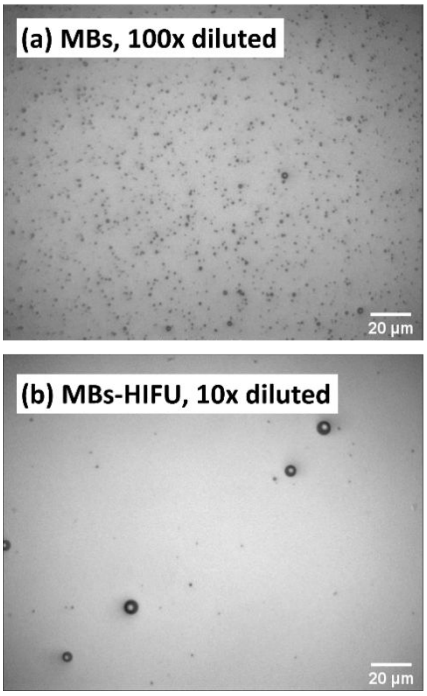

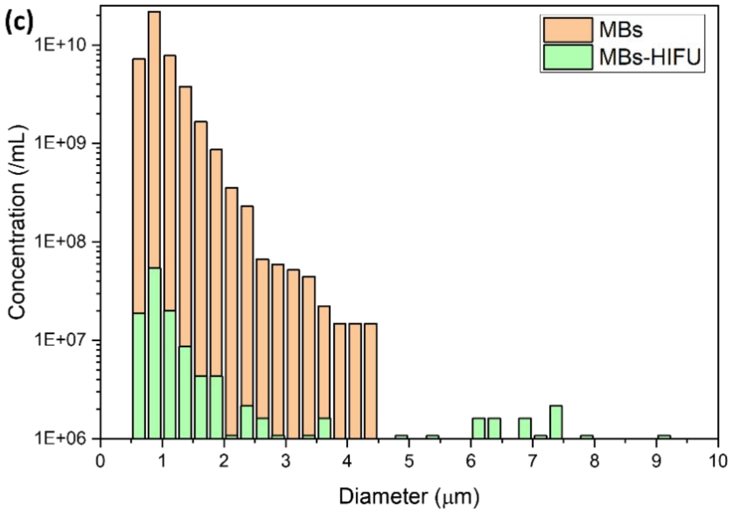

(d)

	Diameter (μm)	Concentration (/mL)
MBs	1.23 ± 0.36	$(4.5 \pm 0.6) \cdot 10^{10}$
MBs-HIFU	2.06 ± 2.10	$(1.4 \pm 0.4) \cdot 10^{8}$

Figure S1 - (a) An optical image of MBs produced by mechanical agitation, the sample was diluted 100x from stock solution. (b) An optical image of the same MB sample after exposure to HIFU, the sample was diluted 10x for imaging. (c) A histogram of MB concentration (MBs/mL) as a function of diameter (μm) for the sample before and after exposure to HIFU. (5) A summary of the average diameter (μm) and concentration (MBs/mL) of the MB sample before and after exposure to HIFU.
